# Primary Intraosseous Mucoepidermoid Carcinoma of the Mandible: A Report of a Rare Case

**DOI:** 10.7759/cureus.115525

**Published:** 2026-08-31

**Authors:** Poornima Chikane, Rasika S Kumavat, Rajiv S Desai, Shivani Bansal, Pankaj M Shirsat

**Affiliations:** 1 Department of Oral and Maxillofacial Pathology, Nair Hospital Dental College, Mumbai, IND

**Keywords:** glandular, intraosseous, mucoepidermoid carcinoma, neoplasm, odontogenic cyst, salivary gland

## Abstract

Mucoepidermoid carcinoma(MEC) is the most common primary salivary gland neoplasm. The exact origin of intraosseous MEC remains uncertain and debated. Although rare, intraosseous MEC is the most common and well-recognized intraosseous salivary gland neoplasm. Histopathological examination revealed cystic and solid epithelial components comprising mucous, epidermoid, and intermediate cells, with strong intracytoplasmic mucicarmine positivity, supporting a diagnosis of low-grade primary intraosseous MEC. The patient subsequently underwent hemimandibulectomy at a higher oncology center and remained disease-free during the available follow-up period. The diagnosis of intraosseous MEC presents a diagnostic challenge due to its clinical and radiological resemblance to various benign odontogenic cysts and tumors. We present an interesting case of intraosseous MEC in a 23-year-old female patient.

## Introduction

Mucoepidermoid carcinoma (MEC), a malignant neoplasm arising from the salivary glands, was initially described by Volkmann in 1895 and later classified as a mucoepidermoid tumor by Stewart and coworkers in 1945 [1]. Primary intraosseous MEC is rare and accounts for only 2% to 4% of all reported cases of MEC. It occurs slightly more commonly in females, and the mandible is affected more often than the maxilla, especially in the posterior region [2].

Proposed mechanisms regarding the pathogenesis and origin of intraosseous MEC include (1) entrapped retromolar mucous glands within the mandible, followed by neoplastic transformation of these glands; (2) ectopically encased embryonic remnants of salivary glands within the mandible; (3) transformation of the lining epithelium of a dentigerous cyst, suggestive of mucous metaplasia; and (4) neoplastic transformation and invasion from the lining of the maxillary sinus [2].

The present case highlights the importance of early diagnosis of indolent yet locally aggressive intraosseous MEC of the mandible, which can mimic benign odontogenic cysts and odontogenic tumors.

## Case presentation

A 23-year-old apparently healthy female patient presented with a history of mild pain and mild swelling in the right mandibular posterior region of the jaw of two months' duration. On intraoral examination, there was a nonpainful swelling with intact overlying mucosa and clinically missing permanent mandibular right second and third molars. No lymphadenopathy or mental nerve paresthesia was observed.

Panoramic imaging revealed a well-circumscribed, multilocular, expansile radiolucency in the right mandibular posterior region associated with submerged permanent mandibular right second and third molars (Figure 1). Keeping the intraosseous location in mind, the differential diagnosis of various odontogenic cysts and tumors, including odontogenic keratocyst, glandular odontogenic cyst, and ameloblastoma, was considered.

**Figure 1:**
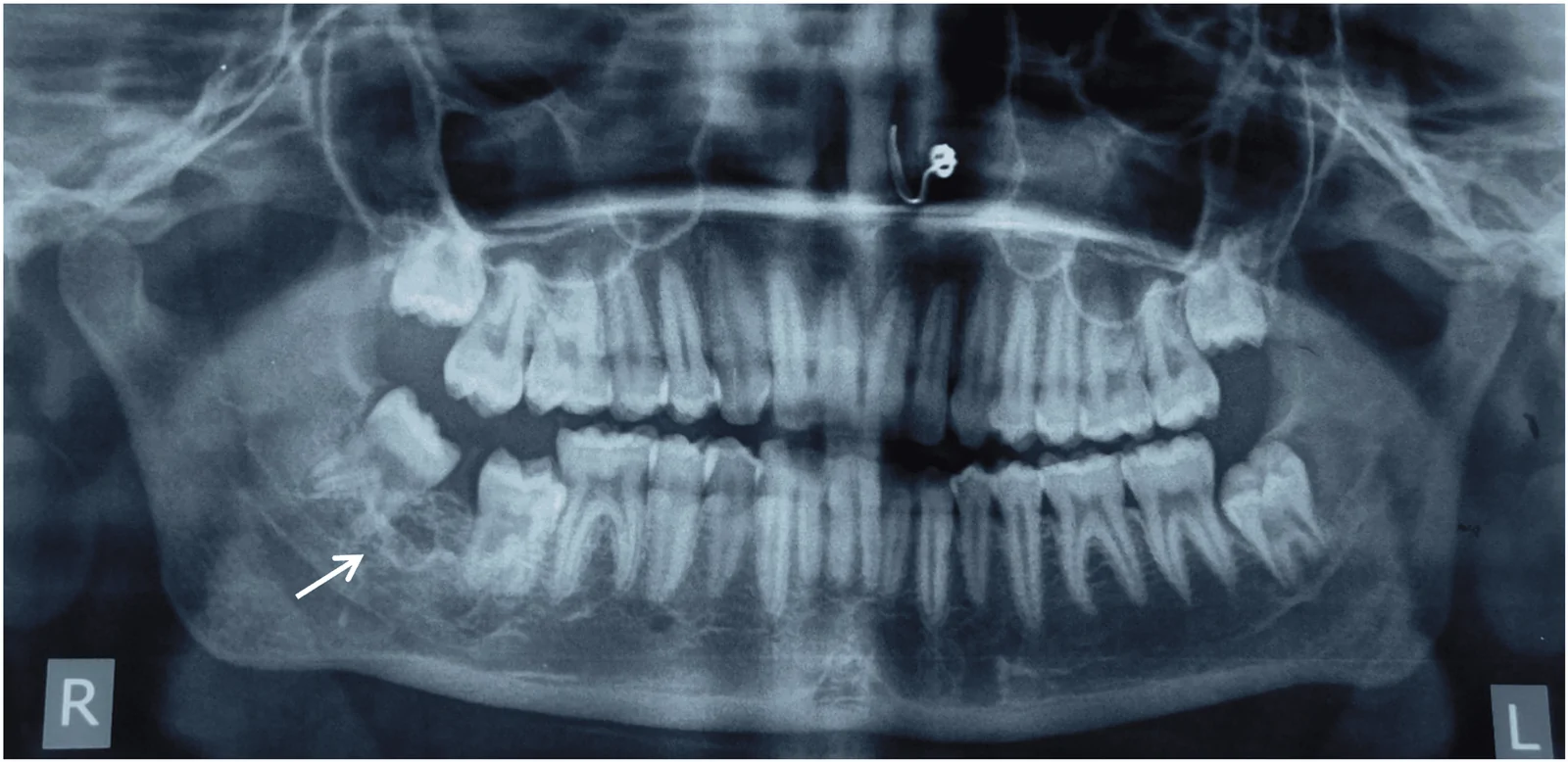
A panoramic radiograph demonstrates a well-defined, multilocular radiolucent lesion associated with impacted mandibular second and third molars; the white arrow indicates the radiolucency associated with the impacted molar on the right side.

Histopathological examination of an incisional biopsy revealed cystic spaces lined by mucous cells with abundant mucinous cytoplasm and eccentric nuclei. The solid epithelial tumor islands composed of mucous, squamoid (epidermoid), and nondescript intermediate cells in variable combinations were evident (Figure 2). The mucicarmine stain revealed strong intracytoplasmic positivity for mucin (Figure 3). The final diagnosis of primary intraosseous low-grade MEC was rendered. The patient was referred to a higher oncology center for further treatment, following which the patient underwent hemimandibulectomy. Currently, we have put the patient on follow-up and the patient is disease-free.

**Figure 2:**
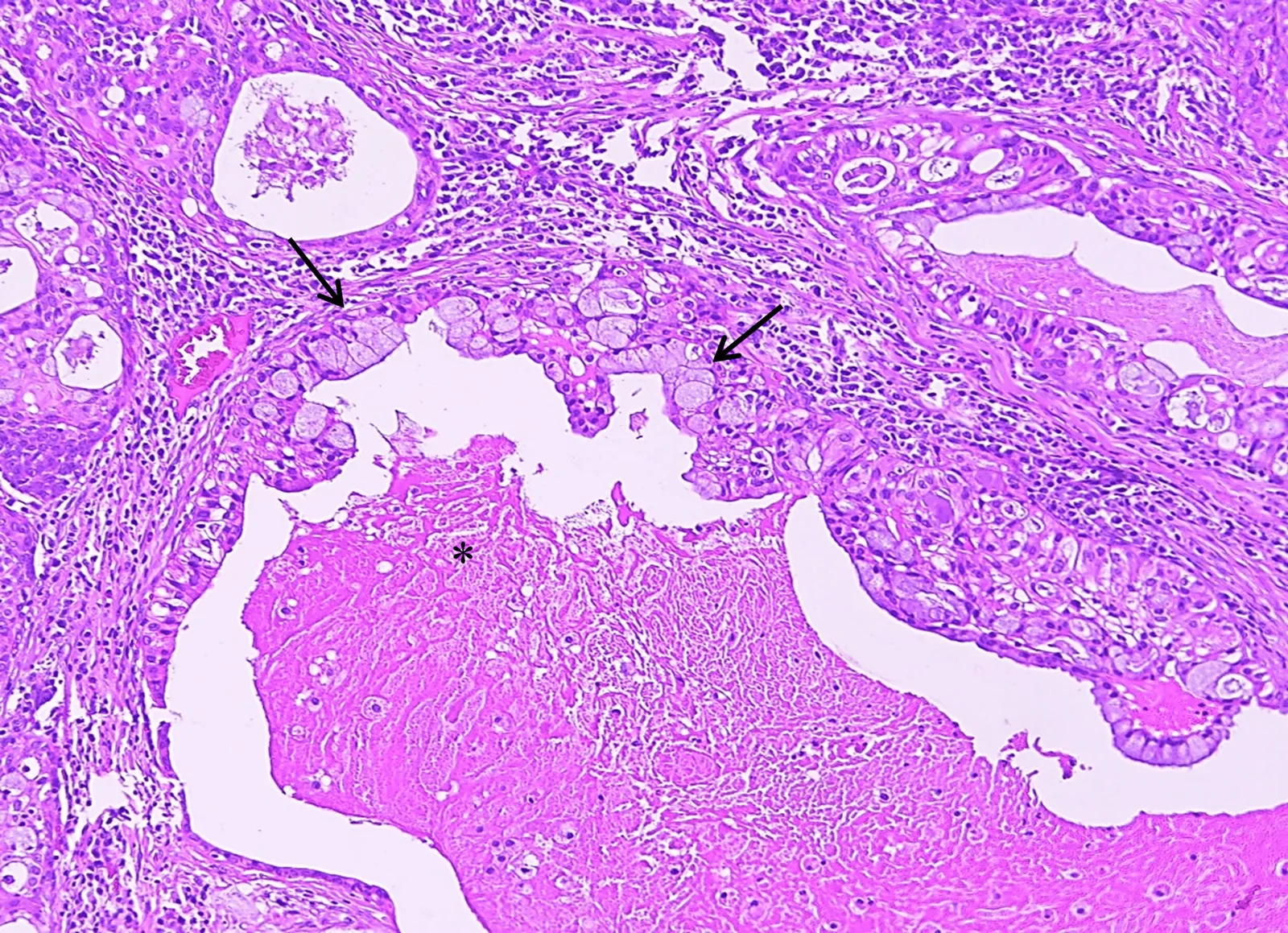
Microscopic image showing cystic spaces lined by squamous epithelium interspersed with columnar mucous cells (arrow) with abundant mucinous cytoplasm (asterisk) (H&E staining, ×10).

**Figure 3:**
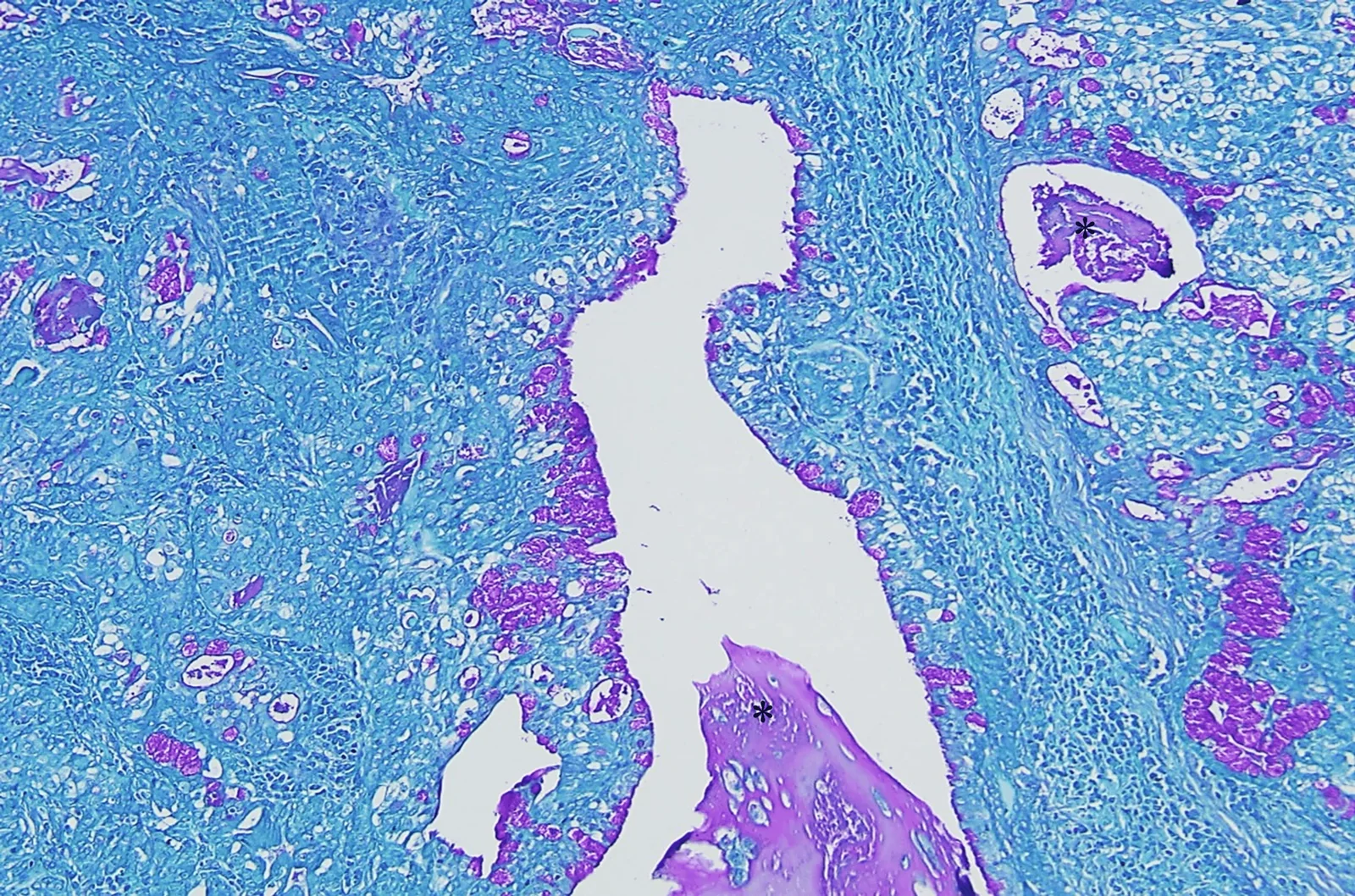
Intracytoplasmic mucicarmine positivity (asterisk) in mucous cells and secretory material within the cystic spaces (mucicarmine staining, ×10).

## Discussion

The primary intraosseous MEC behaves very differently from MECs found in the major and minor salivary glands. Waldron and Mustoe proposed that intraosseous mucoepidermoid carcinoma be considered part of the PIOC classification as type 4 [3]. The WHO defines PIOC of the jaw as a primary malignant tumor of the jaw bone that arises from odontogenic epithelium and has seemingly no connection with the oral mucosa [4]. Intraosseous MEC is usually found twice as frequently in the mandible as in the maxilla and is more frequent in the posterior aspect of the mandible, as evidenced by our low-grade case [5]. Clinically, it commonly presents with swelling, pain, and mandibular numbness; however, cases with conspicuous tooth mobility and dysphagia pertaining to submandibular swelling have also been identified [6]. The present case displayed minimal features with no obvious swelling, similar to the study by de Freitas et al. [5].

Chan et al. demonstrated diverse radiologic features, which are pathognomonic, generally consisting of a well-defined radiolucent lesion lined by a radiopaque sclerotic border interspersed with amorphous internal sclerotic septae. Unilocular intraosseous MEC is often associated with an impacted tooth, mimicking a dentigerous cyst. Evaluation of the panoramic radiograph in our case showed a multilocular radiolucency associated with impacted second and third molars, implying the cystic nature of the lesion. The multiloculated nature of intraosseous MEC necessitates its differentiation from other multilocular cystic lesions, including ameloblastoma, GOC, and odontogenic keratocyst [7].

Brookstone and Huvos [8] formulated a staging system for intraosseous MEC. Lesions with intact cortical plates without bony destruction have an exceptional prognosis and are classified as stage I; on the other hand, lesions with intact plates, showing some degree of intrabony expansion, are interpreted as stage II, and finally, stage III lesions are characterized by cortical perforation or nodal metastasis [7]. Metastasis to regional lymph nodes is uncommon in this rare condition and accounts for 9% of intraosseous MEC, whereas less common sites include the ipsilateral clavicle, lung, and brain [9]. In our case, it should be categorized as stage II, as there was intrabony expansion.

Histologically, mucoepidermoid carcinoma is categorized into three subtypes based on the quantity of mucous, epidermoid, and intermediate cells, the degree of cellular atypia, and the extent of cystic formation. Low-grade tumors typically display prominent cystic structures, minimal atypical features, and a high percentage of mucous cells. In contrast, high-grade tumors are characterized by nests of squamous and intermediate cells exhibiting marked atypia and considerable pleomorphism [10].

The histological and radiological similarities between intraosseous MEC and GOC pose a diagnostic challenge. GOC shares several overlapping microscopic features, including mucous cells, microcysts, duct-like structures, and cystic architecture. Keerthika et al. demonstrated that a positive gene fusion transcript of CRTC1/3::MAML2 expression in intraosseous MEC can be used to differentiate intraosseous MEC from GOC in difficult cases [11].

Radical surgery is the most preferred treatment. A lifelong biannual follow-up examination is warranted for intraosseous MEC. Bell et al. observed significantly lower recurrence rates following segmental or hemimandibulectomy compared with conservative procedures such as curettage or enucleation [2]. Similarly, Simon et al. emphasized that conservative treatment is associated with a greater likelihood of local recurrence because of the infiltrative growth pattern of the tumor [9]. In the present case, the patient underwent hemimandibulectomy and remains disease-free on follow-up, supporting the effectiveness of definitive surgical management.

## Conclusions

A radiolucent lesion of the mandible having septae within the tooth-bearing region encompasses a broad differential diagnosis, reflecting the diverse biological behaviors and tissue origins of jaw pathology. These entities range from benign developmental processes to aggressive malignancies. The present case underscores the clinico-radiopathological correlation for the early diagnosis and management of intraosseous MEC.
